# Elevation of miR-302b prevents multiple myeloma cell growth and bone destruction by blocking DKK1 secretion

**DOI:** 10.1186/s12935-021-01887-y

**Published:** 2021-03-31

**Authors:** Zheyu Wu, Yufeng Zhang, Zhiqiang Yang, Yufan Zhu, Yuanlong Xie, Fuling Zhou, Lin Cai

**Affiliations:** 1 Department of Orthopedics, Zhongnan Hospital of Wuhan University, 169 Donghu Road, Wuhan, China; 2 Department of Hematology, Zhongnan Hospital of Wuhan University, 169 Donghu Road, Wuhan, China

**Keywords:** Multiple myeloma, Myeloma bone disease, miR-302b, DKK1

## Abstract

**Background:**

Myeloma bone disease (MBD) is a severe complication of multiple myeloma (MM) mainly due to an imbalance between enhanced osteoclast activity and reduced osteoblast function. Previous studies have demonstrated that miRNAs play a vital role in the osteogenic differentiation of mesenchymal stromal cells (MSCs) in MM. However, the value of miR‑302b in MBD remains to be further elucidated. The aim of this study is to explore the role of miR‑302b in the regulation of MBD osteogenic differentiation and evaluate the potential of a new therapeutic strategy for the clinical treatment of MBD.

**Method:**

Our previous research demonstrated that MiR-302b belongs to the miR-302 cluster and is able to inhibit tumor growth and osteolysis in an orthotopic osteosarcoma xenograft tumor mouse model. In this study, we first transfected miR-302b mimics, miR-302b inhibitor, and miR-302b NC into MM1.S and RPMI8226 MM cells to detect the correlation between miR-302b expression in the pathological specimens and the clinicopathological features by qPCR, the target correlation between miR-302b and DKK1 by immunohistochemistry, qPCR and Western blot, and the correlation between miR-302b and the Wnt/β-catenin signaling pathway by Western blot. The effect of miR-302b on osteoblastogenesis was also studied in a subperiosteal tumorigenesis model of NOD/SCID nude mice.

**Results:**

We found that increased miR-302b suppressed cell proliferation and induced cell apoptosis in RPMI 8226 and MM1.S cells. TargetScan online bioinformatic analysis predicted that miR-302b is able to bind to 3′UTR of DKK1 mRNA. Target binding of miR-302b to DKK1 was demonstrated by dual-luciferase reporter assay, qPCR, Western blot and immunohistochemistry, indicating that miR-302b is able to degrade DKK1 in RPMI 8226 and MM1.S cells. The model of co-culturing MM cells with preosteoblast MC3T3-E1 cells showed that miR-302b inhibits MM-induced suppression of osteoblast differentiation. Western blotting showed that miR-302b promotes the Wnt/β-catenin signaling pathway in MM cells. Micro-CT and immunohistochemistry results showed that miR-302b suppresses myeloma bone destruction in vivo.

**Conclusion:**

miR-302b is able to target DKK1 and promote the Wnt/β-catenin signaling pathway in MM.

## Background

Multiple myeloma (MM) is an incurable uniformly fatal neoplastic disease originated from B-cell, characterized by malignant clonal hyperplasia of plasma cells in bone marrow(BM), leading to monoclonal immunoglobulin (paraprotein), renal dysfunction, hypercalcemia and lytic lesions in the bone [1, 2]. In addition, myeloma bone disease (MBD) is mainly caused by an imbalance of the bone remodeling process in the BM microenvironment, affecting about 70% of myeloma patients at the time of diagnosis [3]. Bone destruction in MBD is usually attributed to the over-activation of the osteoclastic activity and osteoblastogenesis inhibition [4, 5].

Complex cell–cell interactions between MM tumor cells are essential for the occurrence of MBD [6, 7]. Previous studies reported that MM cells can impair the normal physiological function of BM-derived stromal cells (BMSCs), osteoclasts and osteocytes [8–10]. Overproduction of some cytokines and growth factors secreted by BMSCs including Wnt inhibitors, interleukin-3 (IL-3), tumor necrosis factor alpha (TNF-a) and transforming growth factor b (TGF-b) is believed to be the most important factor contributing to osteoblast dysfunction in MM patients [11–13]. However, the mutual regulation mechanism between MM cells and osteoblasts at different differentiation stages has not been elucidated.

MicroRNAs (miRNAs) are small non-coding single-stranded RNA molecules with about 22 nucleotides which are widely involved in the post-transcriptional regulation of eukaryotic gene expression [14, 15]. Multiple miRNAs including miR-29b, miR-21, miR-15a, miR-192 and miR-215 have been identified to be implicated in the development of MM and the regulation of BM microenvironment [16–19]. A study reported that the overexpression of miR-29b reduces the stimulation response of osteoclasts to RANKL through the target gene c-FOS and the secretion of osteolytic enzymes. Leone et al. showed that miR-21 promotes the progression of MM cells through inhibition of OPG [20]. Furthermore, miRNAs have been shown to be involved in the differentiation process of preosteoblasts. Also, the osteogenesis was reported to be regulated by several miRNAs, including miR-135b, miR-22 and miR-140 [21, 22].

MiR-302b belongs to the miR-302 cluster which is located at the 4q25 chromosome region and serves as a tumor suppressor in osteosarcoma, gastric cancer, hepatocellular carcinoma and bladder cancer [23].Previous studies showed that miR-302b inhibits the tumor growth and osteolysis in an orthotopic osteosarcoma xenograft tumor mouse model established by injecting osteosarcoma cells subperiosteally into the right proximal lateral tibia [24]. However, the role of miR-302b in multiple myeloma and MMD is unclear. We found that the miR-302b expression was reduced in the BM of MM patients compared with that of normal people, and the up-regulation of miR-302b inhibited the proliferation and induced cell apoptosis in MM cell lines and inhibited the MM-induced suppression of osteoblast differentiation. We also revealed that miR-302b can extenuate MM cell-induced bone destruction through targeting DKK1 expression and secretion.

## Materials and method

### Cell culture and osteogenic differentiation

Human multiple myeloma cell lines RPMI8226, and MM1.S were cultured in RPMI1640 supplemented with 10% fetal bovine serum (FBS) at 37 °C in a 5% CO2 atmosphere, and the medium was renewed every three days. MC3T3-E1 cells were cultured in DMEM supplemented with 10% FBS and 1% l-glutamine at 37 °C in a 5% CO2 atmosphere. The cell co-culture model was established through the 24-well multiwell insert System with 1.0 µm pore PET Membrane. MC3T3-E1 cells were seeded in 12-well plates in the lower chamber, and MM cells in the upper chamber. After 24-h co-culture, the MM cells were removed, and MC3T3-E1 were cultured with renewed medium for further study. For osteogenic differentiation, MC3T3-E1 were cultured in α-MEM containing 50uM ascorbic acid 2-phosphate, 5 mM β-glycerophosphate and 100 nM dexamethasone.

### Transient transfection

The MiR-302b mimic and inhibitor were obtained from RiboBio (Guangzhou,China), for whose transfection, MM1.S and RPMI8226 cells at a concentration of 2 × 104 cells/well were incubated in 6-well plates for 24 h. According to the manufacturer’s protocol, the cells were then transfected with 50 nM miR-302b mimic or 100 nM inhibitor in Opti-MEM medium without fetal bovine serum (FBS) addition of Lipofectamine 3000.

### MTT assay

MTT assay was used to detect MM cell viability. MM cells were seeded in 96-well plates at a density of 5000 cells per well in RPMI1640 medium. After 24-h cell seeding, transient transfection was performed according to the manufacturer’s protocol. After 24-h, 48-h and 72-h transfection, 10 µl 5 mg/ml MTT solution was added to each well, and the cells were incubated at 37  °C, 5% CO2 for 4 h and centrifuged at 1000×*g* for 10 min. After removing the medium, 100 μl DMSO was added to each well and the plates were shaken on the oscillator for 10 min until the formazan crystals were dissolved completely. The optical density (OD) of each well was measured at 570 nm using a microplate Reader (PE Enspire).

### Flow cytometry assay

Annexin V-FITC Apoptosis Detection Kit (CA1020) was obtained from Solarbio Science & Technology Co. Ltd (Beijing, China). Briefly, 5 × 105 cells were collected, washed with 4 °C PBS three times, and resuspended in 200 μl Binding Buffer. 10 μl Annexin V-FITC and 10 μl PI were mixed and added into the cell suspension in the darkness to react for 15 min at room temperature. After the addition of 300 μl Binding Buffer, the sample was assayed within 1 h by flow cytometry (Cytoflex, Beckman).

### Alizarin red staining and quantitative analysis of mineralization

After 10-day osteogenic differentiation, MC3T3-E1 cells were washed twice with PBS and then fixed with 4% paraformaldehyde for 5 min. Cell plates were washed with deionized water twice and then stained with 1% Alizarin (pH 4.1) Red S solution for 20 min. The deposited Alizarin Red S solution in each well was collected and mixed with 10% cetylpyridinium chloride (Sigma-Aldrich) and evaluated for absorbance at 560 nm. Then the plates were washed with water three times and viewed under microscope. The orange and red positions were identified as Calcium deposits.

### Quantitative real-time PCR(qPCR)

Total RNA was extracted from cells by using TRIzol Regent (Invitrogen). The RNA was reverse transcribed using a Transcriptor Universal cDNA Master (Roche) according to the manufacturer’s instructions. RT-PCR was performed as the manufacturer’s instructions (Applied Biosystems). PCR amplification was carried out on CFX Connect real-time PCR Detection System(Bio‐Rad, Richmond, CA). Primer sequences: miR-302b forward 5′-ATCCAGTGCGTGTCGTG-3′, reverse 5′-TGCTTAAGTGCTTCCATGTT-3′. U6 was used as an internal control of miR-302b. U6 forward 5′-CTCGCTTCGGCAGCACATATACT-3′, reverse 5′- ACGCTTCACGAATTTGCGTGTC-3′. Other target genes used β‐actin as an internal control. All the experiments were repeated three times.

### Dual‐luciferase reporter assay

The wild‐type (WT) sequence of DKK1 mRNA 3′UTR (WT-DKK1-3′UTR) and mutation (MUT) sequence of DKK1 mRNA 3′UTR with deletion of the miR‐302b binding sites (MUT-DKK1-3′UTR) were designed consequently. Luciferase report vectors were constructed. For dual luciferase reporter detection, miR-302b mimic was co-transfected with WT or mutant pmirGLO-3′UTR vectors into RPMI 8226 or MM1.S cells respectively. After 48-h transfection, the fluorescence activity was detected using Dual‐Glo^®^ Luciferase Assay Reagent (Promega, USA) following the standard protocol on GloMax^®^ 20/20 Luminometer (Promega, USA).

### Western blot detection

Cells were harvested and lysed in the RIPA buffer (Cell Signaling Technology, 9806). After lysate sonication, the protein concentration was determined by BCA assay. Proteins were separated by SDS–PAGE and transferred electrophoretically onto a PVDF membrane (Millipore, IPVH00010). Each antibody incubation was performed overnight at 4 °C followed by the secondary antibody treatment for 1 h at room temperature. Primary antibodies against Bcl-2(CL594-60178, Abcam, 1:5000), Bax (23931-1-AP, Abcam, 1:3000), and DKK1(21112-1-AP, Abcam, 1:3000), Runx2(27000-1-AP, Abcam, 1:3000) and β-catenin (51067-2-AP, Abcam, 1:5000) were used for all analysis, and β-actin (66009-1-Ig, Abcam, 1:2000) was used as an internal control. Immunoblotting was visualized using the ECL system and analyzed using Image-Pro Plus 6.0.

### Establishment of the MM bone destruction mouse model

The animal study was approved by the ethical committee of Zhongnan Hospital of Wuhan University (Wuhan, China). All the procedures for the animals in this study were performed in accordance with the Declaration of Helsinki. MM1.S cells (0.5–1 × 106 cells/100 μl in PBS) were injected into the femur of NOD/SCID (4 weeks old, female) mice, and PBS was injected into the contralateral side as a control. When the mice’s weight dropped significantly, 40 μl hsa-miR-302b agonist (10 mM) with MM of NOD/SCID mice was injected in the femur at a 3-day interval for 2 weeks. Then, the mice were executed and the femurs were isolated for bone destruction assessment by Micro CT. All animal experiments were performed with approval from the Animal Study Committee of Wuhan University and conformed to the relevant guidelines and legislation.

### Micro CT detection

Micro-CT analysis was performed using R_mCT2 (SkyScan1278, Bruker) with an isometric resolution of 40 μm, or ScanXmateL090 with an isometric resolution of 12 μm. The micro-CT files were reconstructed as TIFF images and transferred to TRI/FCS-BON for quantitative analysis. The mineralized tissue volume was measured using a calibration curve obtained from MicroCT Bar Pattern NANO Phantom.

### Histology and Immunohistochemistry

Histological analysis of induced osteoblastic lesions was performed by H&E and von Kossa staining using paraffin and undecalcified sections respectively. We performed ALP staining with the BCIP/NBT Alkaline Phosphatase Color Development Kit (Beyotime Biotechnology, Shanghai, China) and Alkaline Phosphatase Assay Kit (Beyotime Biotechnology, Shanghai, China) to quantify ALP activity. We fixed cells with 4% paraformaldehyde for 15 min at room temperature after 7 days of osteogenic induction, and then the ALP staining was performed according to the instructions. The reaction system was incubated at 37 °C for 10 min and the values of absorbance were measured by Varioskan LUX microplate reader (Thermo Fisher Scientific). We conducted ARS staining to detect the mineralized nodules after 14 days of osteogenic differentiation. All the ARS staining was performed using Alizarin Red S Stain Solution (Cyagen, China). Immunohistochemistry was performed on frozen sections as mentioned previously. Sections were examined using a fluorescence microscope (TE2000U + NT88, NIKON) or a confocal microscope (μSurf, Nanofocus).

### Statistical analyses

All the data were presented as the mean ± SEM. Parametric statistical analysis was performed by Student’s t-test and one-way ANOVA with Tukey’s HSD test. Nonparametric statistical analysis was performed by Mann–Whitney U test, or Kruskal–Wallis test using R v3.3.1. The values were considered significant at P < 0.05. The results were representative out of more than three individual experiments.

### Clinical MM specimens

The study was approved by the Ethics Committee of Zhongnan Hospital of Wuhan University and conducted in accordance with the Declaration of Helsinki. Twelve pairs of bone marrow were collected from the MM patients and healthy individuals by the Department of Orthopedics of Zhongnan Hospital of Wuhan University from February 2019 to July 2020. All the specimens were obtained with patients’ informed consent. All tumor samples were pathologically diagnosed as MM according to the World Health Organization (WHO) Classification of myeloid and lymphoid neoplasms.

## Result

### Elevation of miR-302b suppresses cell proliferation and induces the cell apoptosis in RPMI 8226 and MM1.S cells

**Fig. 1:**
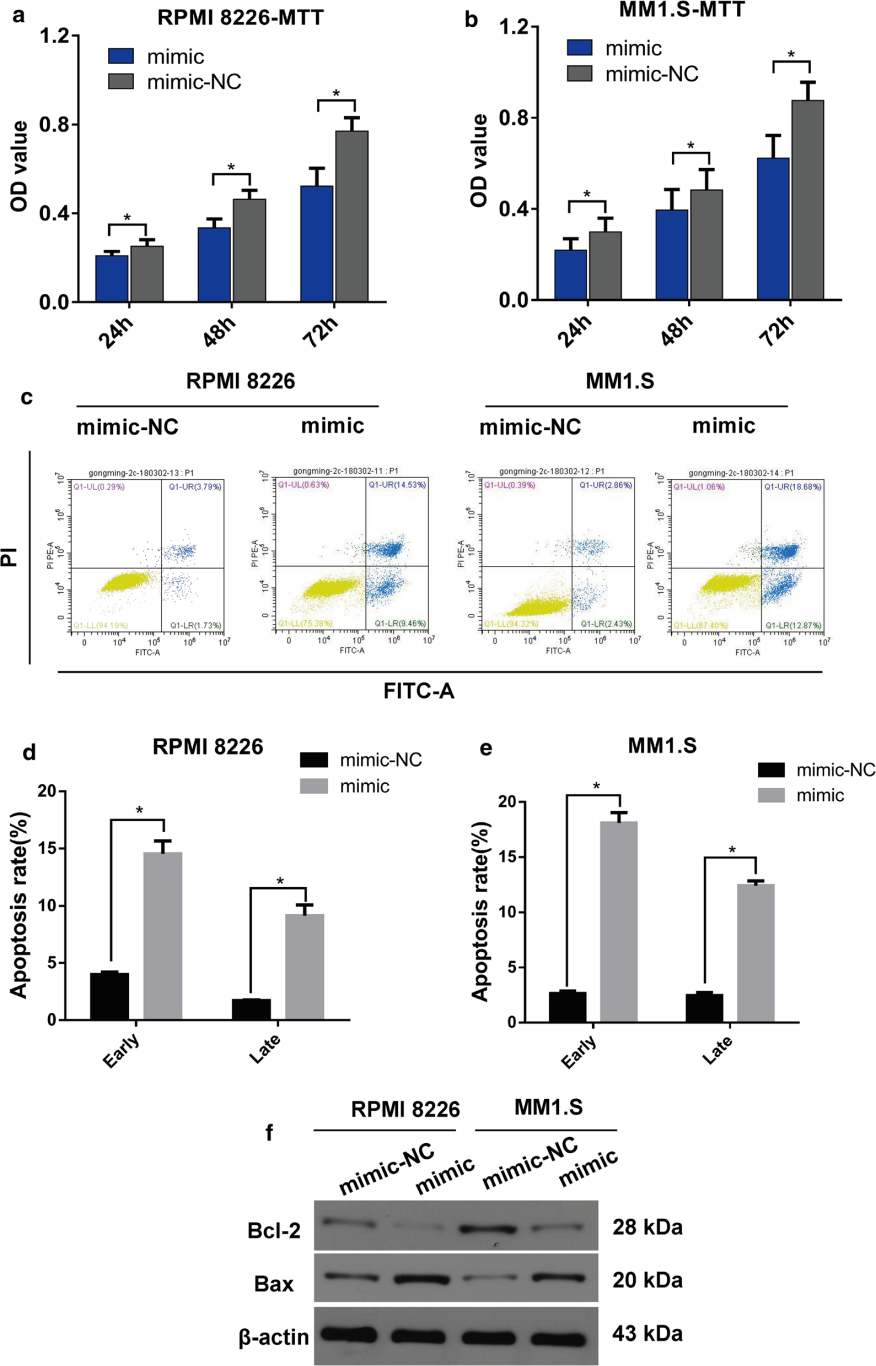
Overexpression of miR-302b suppressed cell proliferation and promoted apoptosis in vitro. **a**, **b** OD value of RPMI 8226 and MM1.S cells was measured by MTT assay at 24, 48, 72 h after the transient transfection of the miR-302b mimic. **c** Representative images of the apoptosis detection. **d** Cartogram of the apoptosis rate of cells in each group. **e** Expression of the relative protein in RPMI 8226 and MM1.S cells

To explore the effect of miR-302b on MM, we collected BM samples from 12 MM patients and 12 healthy individuals. The results showed that the miR-302b expression in BM of the MM patients is lower than that in healthy subjects. MiR-302b in RPMI 8226 and MM1.S cell lines were elevated after transient transfection of the miR-302b mimic (Fig. 1a–c). MTT assay results showed that increased miR-302b significantly suppresses cell proliferation at 24, 48 and 72 h after transient transfection of the miR-302b mimic in RPMI 8226 and MM1.S cells. The apoptotic rate in the early and late stages was significantly increased in miR-302b mimic group compared with that in the control group in RPMI 8226 and MM1.S cells (Fig. 1d, e). The relative protein expression of Bcl-2 was increased, and Bax was inhibited in miR-302b mimic group compared with the control group in RPMI 8226 and MM1.S cells (Fig. 1f). The gain-of-function study showed that increased miR-302b expression suppresses cell proliferation in RPMI 8226 and MM1.S cells due to cell apoptosis induced by miR-302b.

### Inhibition of miR-302b promotes cell proliferation and arrests the cell apoptosis in RPMI 8226 and MM1.S cells

**Fig. 2:**
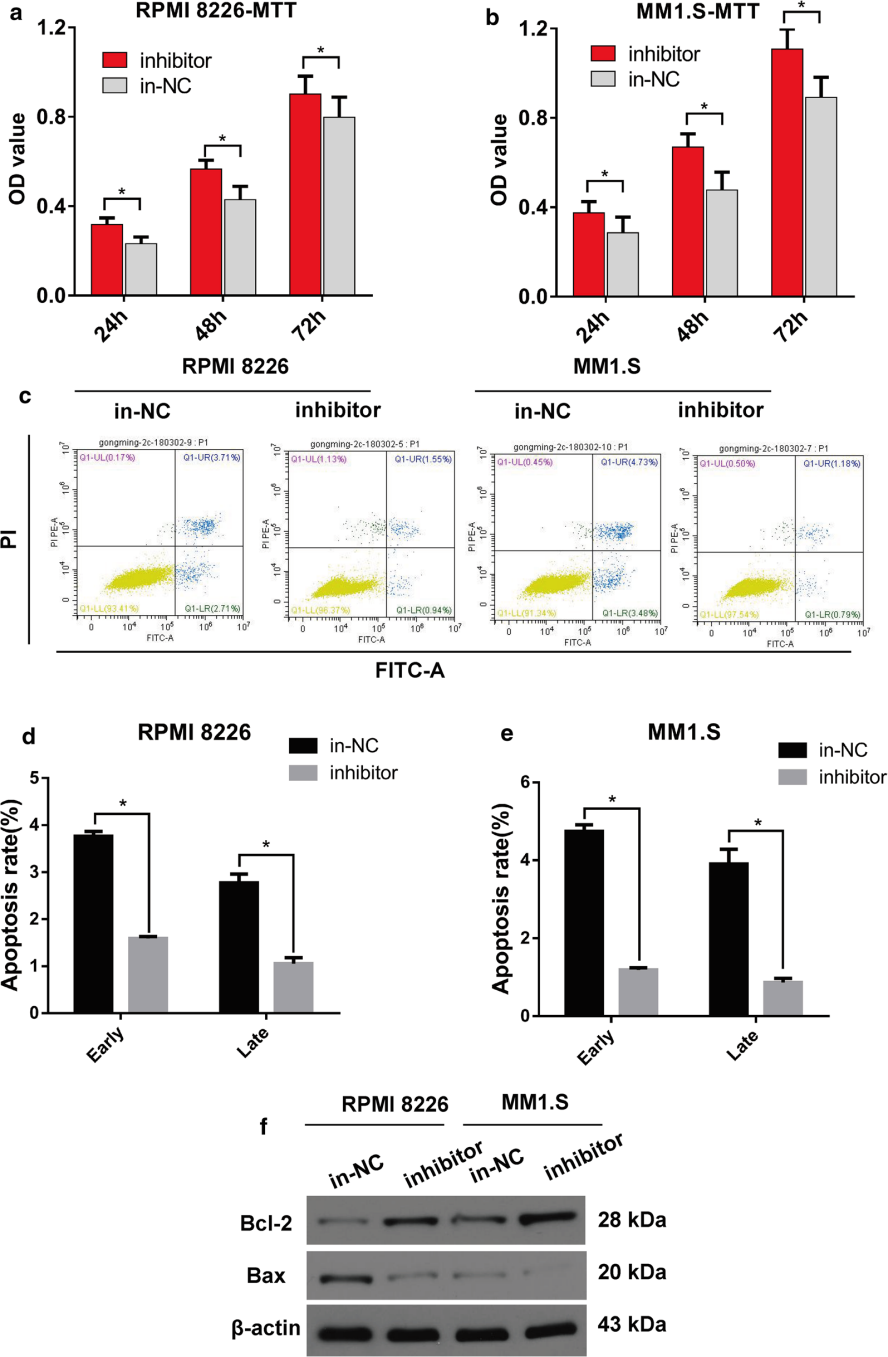
Inhibition of miR-302b promoted cell proliferation and suppressed apoptosis in vitro**. a**, **b** OD value of RPMI 8226 and MM1.S cells was measured by MTT assay at 24, 48 and 72 h after the transient transfection of miR-302b inhibitors. **c** Representative images of the apoptosis detection. **d** Cartogram of the apoptosis rate of cells in each group. **e** Expression of the relative protein in RPMI 8226 and MM1.S cells

For the purpose of evaluating the loss-of-function study on miR-302b in RPMI 8226 and MM1.S cells, miR-302b was down-regulated after transient transfection with the miR-302b inhibitor. The promoting rate of RPMI 8226 and MM1.S cell proliferation at 24, 48 and 72 h after transient transfection in the miR-302b inhibitor group was compared with that in the control group (Fig. 2a–c). Both early-stage and late-stage apoptosis rates significantly decreased in the miR-302b inhibitor group compared with those in the control group in RPMI 8226 and MM1.S cells (Fig. 2d, e). Suppression of miR-302b inhibited the relative protein expression of Bcl-2 and promoted Bax in RPMI 8226 and MM1.S cells (Fig. 2f). The loss-of-function study showed that suppression of miR-302b promotes cell proliferation in RPMI 8226 and MM1.S cells.

### miR-302b targets DKK1 in RPMI 8226 and MM1.S cells

**Fig. 3:**
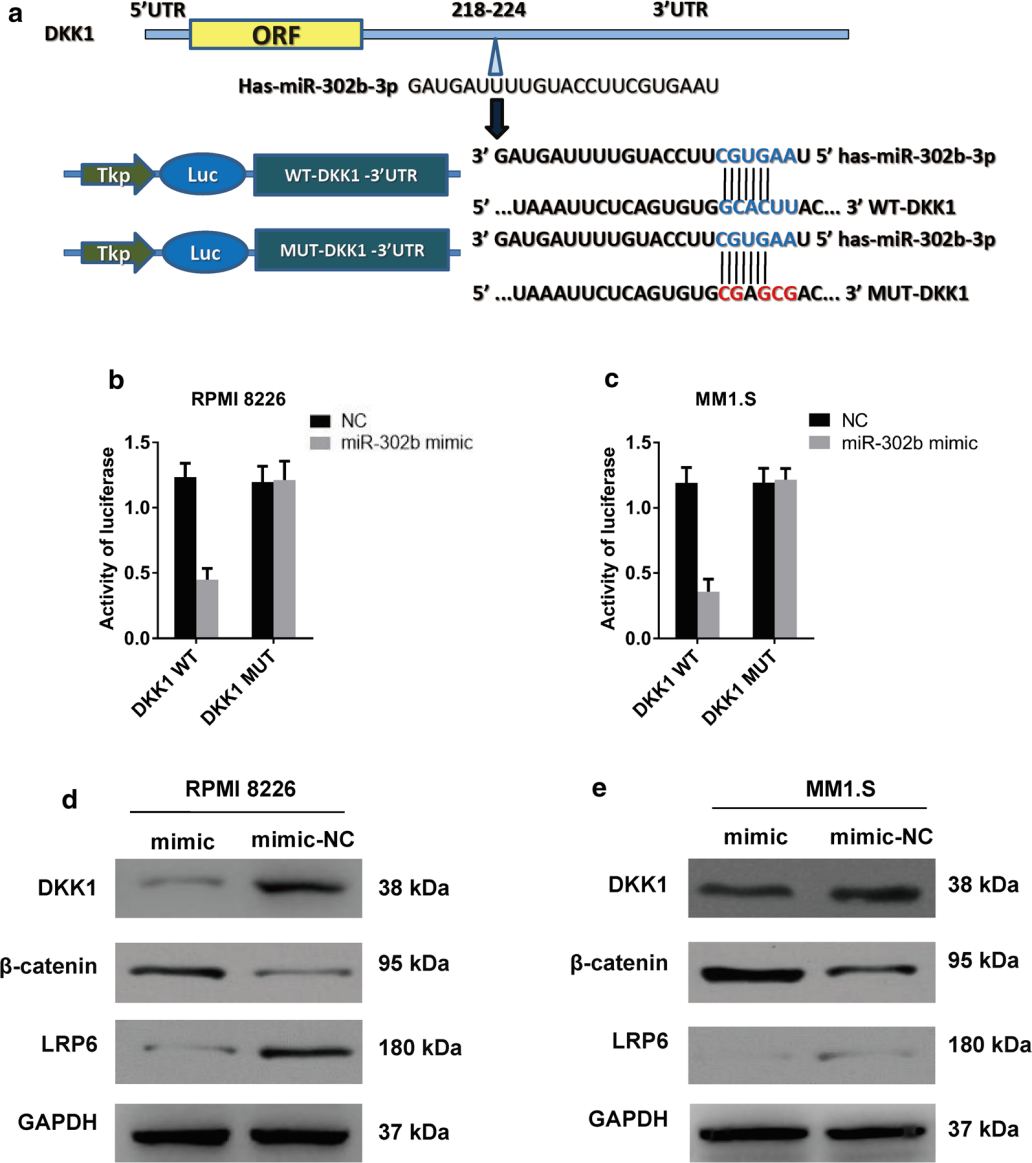
DKK1 as a direct target of miR-302b in MM cells. **a** Sequence alignment of predicted miR-302b binding sites within the DKK1 3′UTR and its mutated sequence for luciferase reporter assay. **b**, **c** Luciferase reporter assay was performed in RPMI 8226 cells and MM1.S cells that were co-transfected with miR-302b mimics and reporter vectors containing DKK1 3′UTR or mutated DKK1 3′UTR. Relative luciferase activity is presented. *P < 0.05 vs. NC group. Data are presented as mean ± SD from three separated experiments. **d**, **e** Expression of DKK1 in RPMI 8226 and MM1.S cells after the transfection of miR-302b mimic and mimic control

Targetscan online bioinformatic analysis predicted that miR-302b can bind to 3′UTR of DKK1 mRNA. A dual-luciferase reporter assay was applied to clarify whether the 3′-UTR of DKK1 mRNA was directly targeted by miR-302b. The putative WT binding site of DKK1 mRNA 3′-UTR was cloned into the pmirGLO vector. For dual luciferase reporter detection, the miR-302b mimic was co-transfected with the WT or mutant pmirGLO-3′UTR vector into RPMI 8226 or MM1.S cells (Fig. 3a). The relative luciferase activity was significantly suppressed after miR-302b mimic transfection in the presence of WT 3′-UTR of DKK1 in RPMI 8226 or MM1.S cells (Fig. 3b, c), whereas the miR-302b mimic showed no significant change in luciferase activity in RPMI 8226 and MM1.S cells co-transfected with mutant type 3′-UTR of DKK1. In addition, the miR-302b mimic inhibited the protein expression of DKK1 in RPMI 8226 and MM1.S cells (Fig. 3d, e). These results suggested that miR-302b can target 3′-UTR of DKK1 mRNA in RPMI 8226 and MM1.S cells.

### miR-302b inhibits MM-induced suppression of osteoblast differentiation

**Fig. 4:**
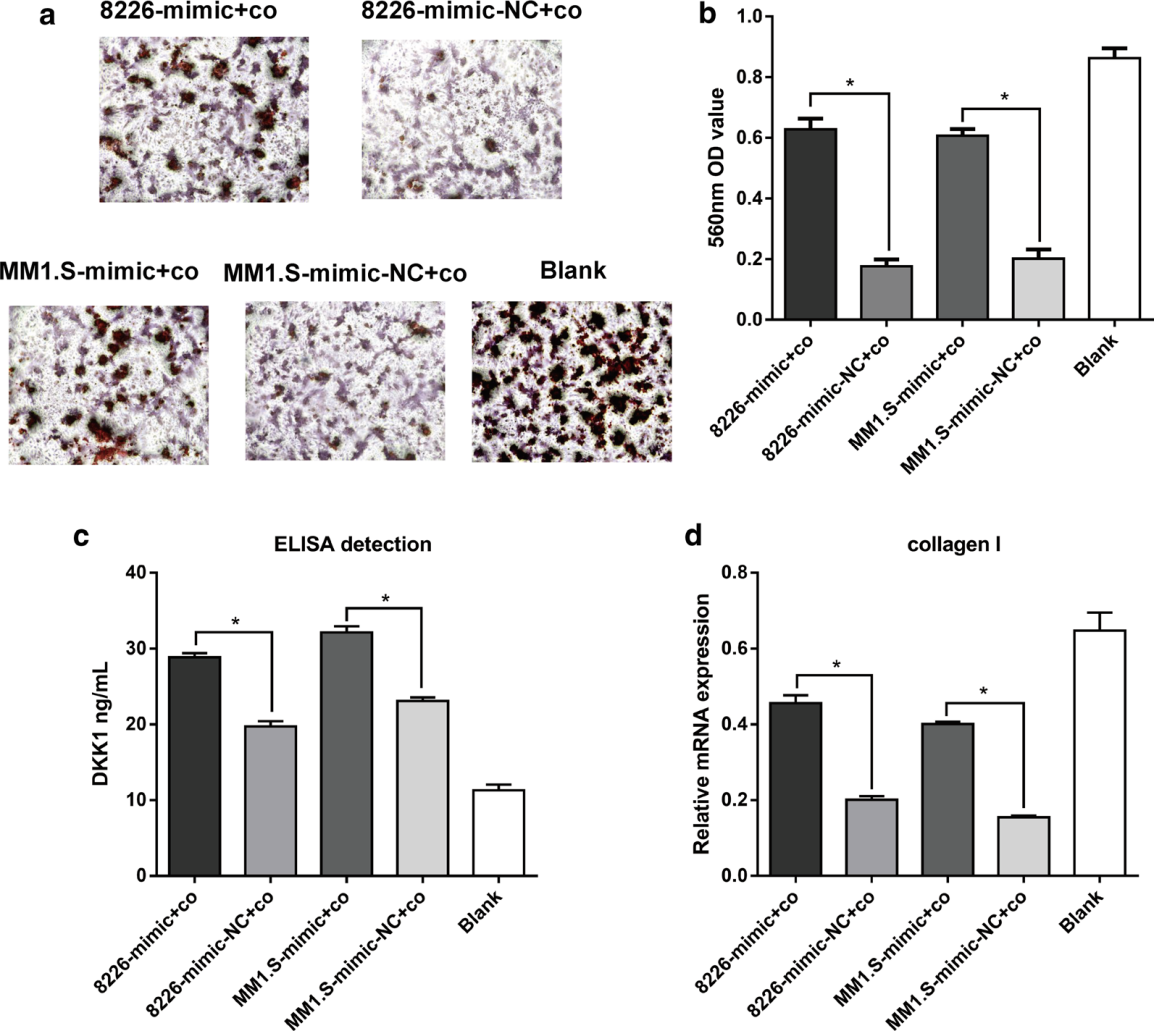
Effects of miR-302b on MM-induced osteogenic differentiation. **a** Alizarin red staining of MC3T3-E1 cells co-cultured with RPMI 8226 or MM1.S after transfection with miR-302b mimics and miR-302b mimics-NC respectively. **b** Quantification of the absorbance at 560 nm in **a** groups. **c** DKK1 protein content in the supernatant of MC3T3-E1 cells in **a** groups. **d** Collagen I mRNA expression of MC3T3-E1 in **a** groups. Data are expressed as mean ± SD. *, P < 0.05

A model of co-culturing MM cells with preosteoblast MC3T3-E1 cells was applied to further elucidate the potential role of miR-302b in MM bone disease. Co-culture with RPMI 8226 or MM1.S suppressed the mineralization capacity of MC3T3-E1 cells compared with the cells without co-culture in the blank group (Fig. 4a, b). The mineralization capacity of MC3T3-E1 co-cultured with miR-302b up-regulated RPMI 8226 or MM1.S was stronger than that of MC3T3-E1 co-cultured with miR-302b normally expressed MM cells. DKK1 protein in the supernatant of MC3T3-E1 cells was detected by ELISA. Both RPMI 8226 and MM1.S promoted the DKK1 protein content in the supernatant of MC3T3-E1 at 24 h after co-culture compared with the blank control group (Fig. 4c). Furthermore, DKK1 protein content in the supernatant of MC3T3-E1 co-cultured with miR-302b up-regulated RPMI 8226 or MM1.S was increased compared with the control group. RT-PCR results showed that both RPMI 8226 and MM1.S suppresses the mRNA expression of Collagen I of MC3T3-E1 at 24 h after co-culture. The Collagen I mRNA expression of MC3T3-E1 cells co-cultured with miR-302b was lower in RPMI 8226 or MM1.S than that of MC3T3-E1 cells co-cultured with miR-302b and normally expressing MM cells (Fig. 4d). Taken together, these results proved that MM cells can impair osteogenic differentiation of MC3T3-E1 cells, and miR-302b can reverse the MM-induced suppression of MC3T3-E1 cell differentiation.

### miR-302b inhibits the secretion of DKK1 in MM cells and promotes the Wnt/β-catenin signaling pathway in MC3T3-E1 cells

**Fig. 5:**
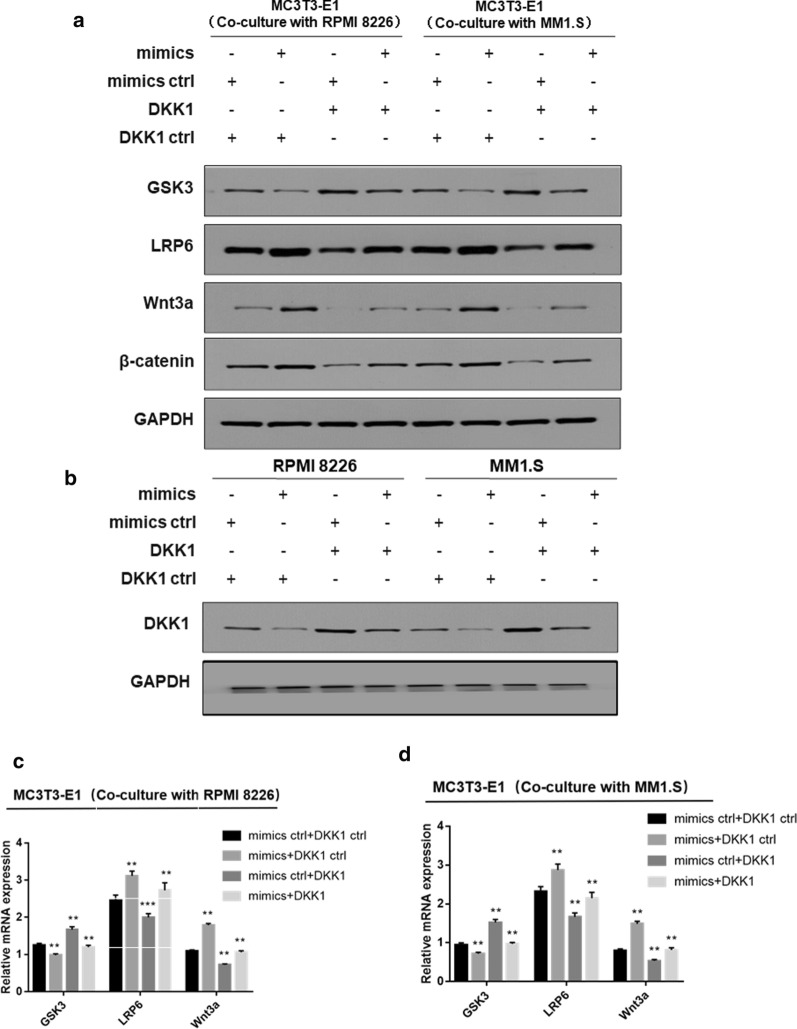
miR-302b inhibits the secretion of DKK1 in MM cells and promotes the Wnt/β-catenin signaling pathway in MC3T3-E1 cells. **a**, **b** The expression levels of key proteins of the Wnt/β-catenin pathway in MC3T3-E1 cells co-cultured with RPMI 8226 or MM1.S were detected by Western blotting. RPMI 8226 and MM1.S cells were transfected with miR-302b mimics (mimics)/miR-302b mimics-control (mimics ctrl) and DKK1 overexpression vectors (DKK1)/negative control vectors (DKK1 ctrl). **c**, **d** Expression of relative protein of the Wnt/β-catenin pathway in MC3T3-E1 cells

It is generally known that wnt/β-catenin signaling pathway plays an important role in the development of bone. The antagonistic protein DKK1 is also involved in many processes in bone development and metabolism. To better understand whether the Wnt/β-catenin signaling pathway was involved in the regulation of osteogenic differentiation in MC3T3-E1 cells, the miR-302b mimic (mimic)/miR302b mimic-control (mimic ctrl) and the DKK1 overexpression vector (DKK1)/negative control vector (DKK1 ctrl) were cotransfected into RPMI 8226 and MM1.S cells. We set up a system of co-culture between MC3T3-E1 cells and RPMI 8226/MM1.S as the previous work. The relative protein expression markers were detected by Western blot. The expression of DKK1 with miR-302b up-regulated was decreased in RPMI 8226 and MM1.S cells as compared with the control group, and vice versa. GSK3, LRP6, Wnt3a and β-catenin were well established as the key regulator of the Wnt/β-catenin signaling pathway. The experiment results showed that the expression of LRP6 and Wnt3a was markedly up-regulated in MC3T3-E1 cells when miR-302b overexpressed in RPMI 8226 and MM1.S cells (Fig. 5a, b). Moreover, over expression of DKK1 reduced WNT3a and β-catenin expression in MC3T3-E1 cells compared with the corresponding control cells (Fig. 5a, b). These findings indicated that WNT3a and β-catenin might be important downstream targets of the DKK1 cascade. These results demonstrated that the Wnt/β-catenin signaling pathway in MC3T3-E1 cells is inhibited by DKK1, which secreted by MM cells. In addition, miR-302b can reduce the secretion of DKK1 by MM cells, and promotes the Wnt/β-catenin signaling pathway in MC3T3-E1 cells.

### miR-302b suppresses myeloma bone destruction in vivo

**Fig. 6:**
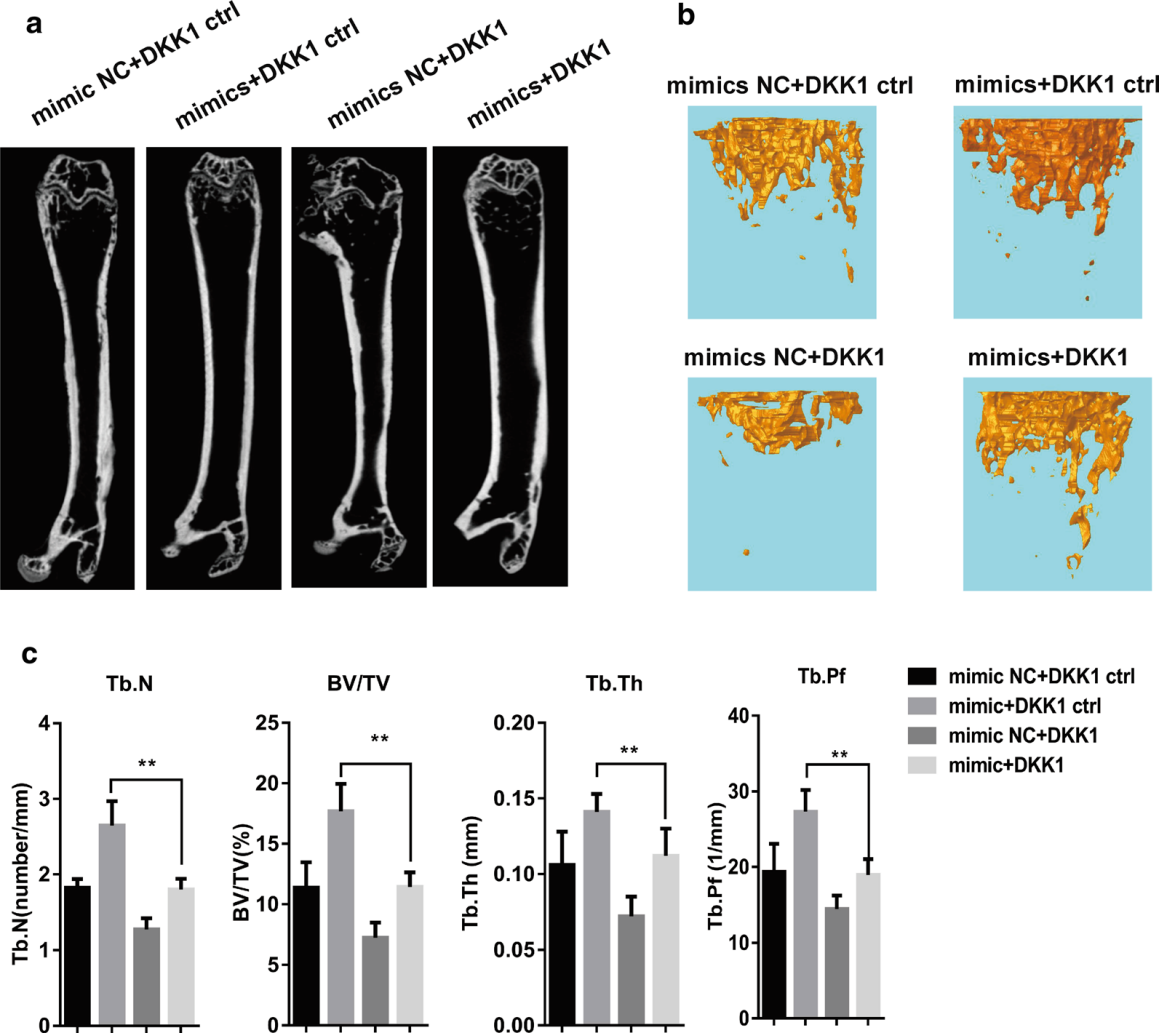
miR-302b delivery prevented MM-induced bone loss. **a**, **b** The femur images of micro-CT scanning of the NOD/SCID nude mice of each group. **c** Histomorphometric analysis of the trabecular bone in vertebrae, including BV/TV, Tb.Th, Tb.N, Tb.Pf Mean§SD, n = 8 biologically in dependent samples,* *P < 0.01by two-way ANOVA

For the purpose of investigating whether miR-302b suppresses MM cell-induced bone destruction in vivo, MM1.S cells transfected with the miR-302b mimic or miR-302b negative control were injected into the femur of the NOD/SCID nude mice. Eight weeks after injection, the mice were executed and the femurs were detached for micro-CT detection. We found that the bone volume and the trabecular number in the mouse femur increased in the miR-302b mimic group compared with miR-302b negative group (Fig. 6a). According to the immunohistochemistry results, the DKK1 expression intensity in miR-302b mimic transfection group was lower than that in the miR-302b negative control transfection group, suggesting that miR-302b overexpression suppresses MM bone destruction in vivo. Four weeks after the molding operation, DKK1 recombinant protein was locally injected into the femur of the MM bone destruction modeled mice induced by MM1.S cell transfection with the miR-302b mimic. Micro-CT results showed that DKK1 offsets the suppression of the miR-302b mimic in terms of the bone volume and trabecular number in the mouse femur (Fig. 6b, c). Furthermore, DKK1 recombinant protein enhances the loss-of-bone volume and trabecular number in NC DKK1 mimic group compared with the control group.

### Down-regulated miR-302b is associated with an increased DKK1 expression in the bone marrow of MM patients

**Fig. 7:**
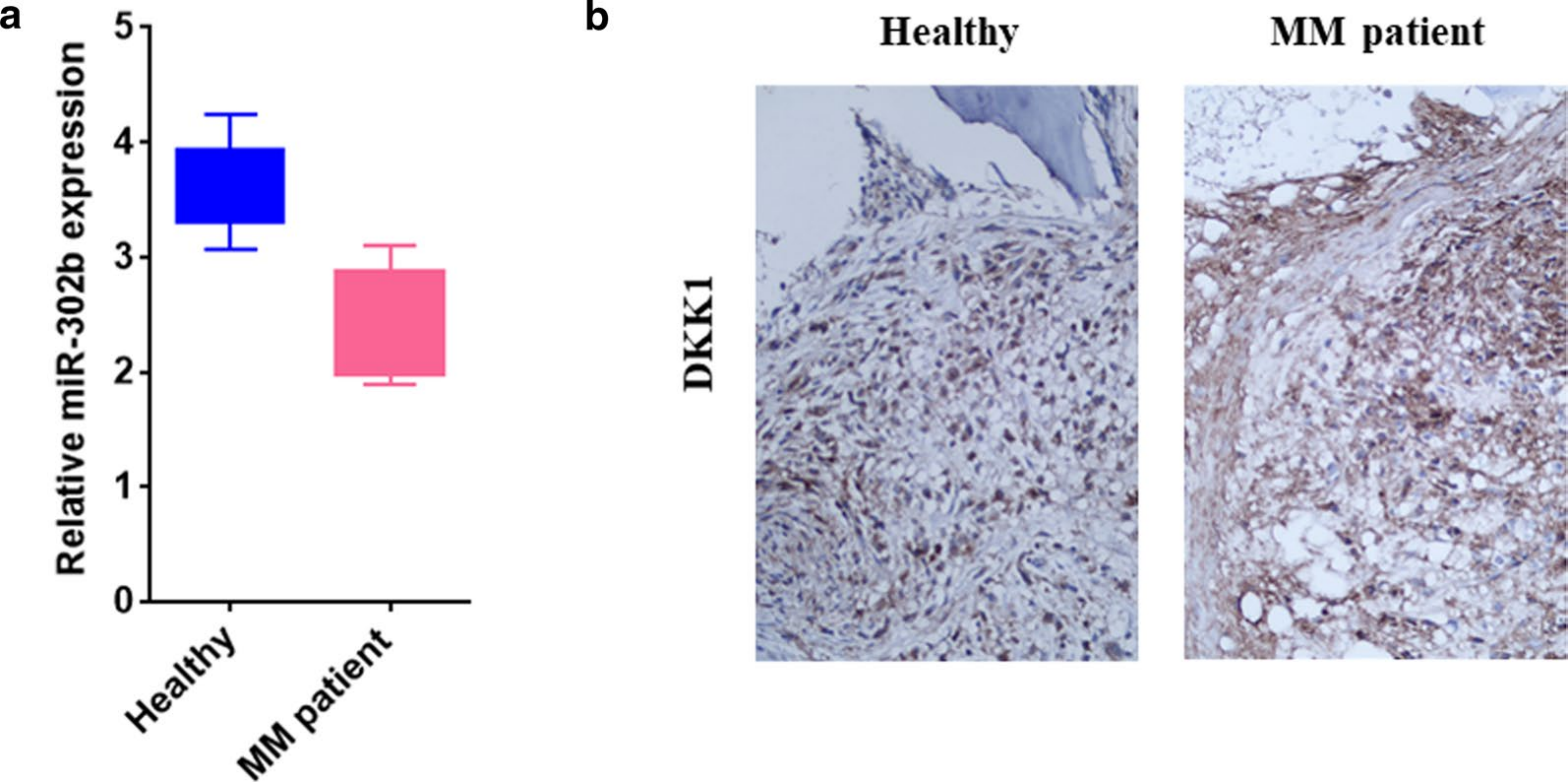
The miR-302b expression in clinical MM specimens. **a** Relative expression of miR-302b in MM tissues vs healthy group. **b** Immunohistochemical staining of DKK1 in MM tissues and paired normal tissues

Twelve pairs of BM collected from MM patients were detected by RT-PCR, and we found that miR-302b expression was decreased in BM of the MM patients compared with that in healthy individuals. The immunohistochemistry results showed that the DKK1 expression intensity was increased in BM of the MM patients compared with that in healthy individuals (Fig. 7a, b). These results demonstrated that up-regulated mir-302b can inhibit DKK1 expression significantly in healthy individuals, and the down-regulated of miR-302b causes an increased DKK1 expression in the bone marrow of MM patients. The findings lend credence to previous studies in vitro and in vivo.

## Discussion

MBD is a progressive disease characterized by osteolysis, pathological fracture, osteoporosis and intractable bone pain. In MBD, physiological coordination between osteoblasts and osteoclasts is destroyed by invasive MM cells, resulting in osteogenic-osteoclast coupling imbalance [25]. It is therefore essential to gain insights into the mechanism underlying the interaction between MM cells and preosteoblasts within the BM microenvironment. Several miRNAs are believed to be involved in the regulation of MM cell proliferation and osteogenic differentiation of preosteoblasts. However, the role of miRNAs in MBD has not been completely uncovered at present.

The present study demonstrated that the miR-302b expression decreases in BM of MM patients, and overexpressing miR-302b can impede cell proliferation and induce MM cell apoptosis. Both RPMI 8226 and MM1.S suppresses osteogenic differentiation of preosteoblast MC3T3-E1. Overexpression of miR-302b in MM cells offsets the inhibition of MM cells on osteogenic differentiation of preosteoblast MC3T3-E1. In addition, MM cells overexpressed with miR-302b can reduce bone volume loss and the trabecular number compared with MM cells with normal miR-302b expression. Previous studies showed that miR-302b expression is reduced in osteosarcoma, liver cancer and prostate cancer, and increased expression of miR-302b can inhibit the biological activity of these cancer cells [23, 26, 27]. It was reported that the expression level of miR-221-5p is significantly lower in MBD-MSCs compared with that in N-MSCs, and knocking down miR-221-5p promotes osteogenic differentiation in MBD-MSCs [25]. Thus miR-302b can suppress MM cell proliferation and MM cell-induced bone destruction.

A dual-luciferase reporter assay demonstrated that miR-302b targets 3′-UTR of DKK1 mRNA in MM cells. MiR-302b also inhibits the secretion of DKK1 in MM cells. DKK1 is a secreted protein that binds to the Wnt receptor LRP5 on the cell membrane and inhibits the Wnt/β-catenin signaling pathway associated with osteogenic differentiation. DKK1 expression in BM and the serum of MM patients with osteolytic destruction is significantly higher than that of MM patients without osteolytic destruction, and the expression level of DKK1 is correlated with the degree of osteolytic destruction as shown by MR imaging of MM patients. Tian et al. found that the anti-DKK1 vaccine triggers CD4+ and CD8+ T cell responses to protect nude mice against MM invasion. Therefore, miR-302b can rescue the inhibition of MM cells on osteogenic differentiation of MC3T3-E1 cells through suppressing DKK1 secretion.

As shown by a series of experiments, miR-302b is downregulated in MM, and overexpression of miR-302b impedes MM proliferation and metastasis both in vitro and in vivo. DKK1 as one of the four members of the extracellular Wnt inhibitors family may prove to be a potential target of miR-302b in MM.

## Conclusion

We demonstrated that miR-302b plays an important role in the development of MM, and that miR-302b overexpression inhibits MM cell growth and bone destruction by down-regulating DKK1 secretion. Our findings contribute to a better understanding of how miR-302b modulates the Wnt/β-catenin signaling pathway in MM, and may provide a potential prognostic marker and a therapeutic target for the treatment of MM.

## Data Availability

All the data and materials were available under the agreement of the authors
